# Effect of simulated warming on the functional traits of *Leymus chinensis* plant in Songnen grassland

**DOI:** 10.1093/aobpla/plz073

**Published:** 2019-11-08

**Authors:** Rui Guo, Ji Zhou, Xiuli Zhong, Fengxue Gu, Qi Liu, Haoru Li

**Affiliations:** 1 Key Laboratory of Dryland Agriculture, Institute of Environment and Sustainable Development in Agriculture, Chinese Academy of Agricultural Sciences, Beijing, P.R. China; 2 Land Consolidation and Rehabilitation Centre, Ministry of Natural Resources of the People’s Republic of China, Beijing, P.R. China

**Keywords:** Carbon, growth, *L. chinensis*, nitrogen, physiology, warming

## Abstract

*Leymus chinensis* grassland in Northeast China provides a natural laboratory for the investigation of climate change. The response of *L. chinensis* to experimental warming can provide insight into its regeneration behaviour and the likely composition of future communities under warmer climate. We used MSR-2420 infrared radiators to elevate temperature and examined soil organic carbon and nitrogen and soil total phosphorus and determined the growth and physiology of *L. chinensis* in response to manipulations of ambient condition and warming. Results showed that compared with the control, *L. chinensis* subjected to warming treatment showed increased soil organic carbon and soil total nitrogen, but no significant difference was observed in soil total phosphorus. Climate warming increased shoot biomass, ecosystem respiration, and ecosystem water-use efficiency and reduced net ecosystem CO_2_ exchange and evapotranspiration. This result implies that warming could rapidly alter carbon fluxes. The effect of warming treatment significantly increased the contents of glucose and fructose and significantly inhibited sucrose synthesis. However, the *TCA* cycle was enhanced when citric and malic acid contents further accumulated. The results implied that *L. chinensis* probably enhanced its warming adaption mechanism mainly through increasing glycolysis consumption when it was exposed to elevated temperature. These results provide an understanding of the fundamental evidence explaining the primary metabolism of *L. chinensis* in response to warming and suggest the future impact of the terrestrial carbon-cycle feedback on global climate change.

## Teaser

Understanding how grasses are affected by a long-term increase in temperature is crucial to understanding the future impact of global climate change. Measurements of soil properties, ecosystem gas exchange and physiological characteristics were made in *Leymus chinensis* grassland under elevated temperatures. The result showed that warming increased the soil organic carbon, soil total nitrogen, shoot biomass, ecosystem respiration and ecosystem water-use efficiency, but reduced net ecosystem CO_2_ exchange and evapotranspiration. This result implies that warming could rapidly alter carbon fluxes, and *L. chinensis* probably enhanced its warming adaptation mechanism mainly through increasing glycolysis consumption.

## Introduction

Climate change increased production of greenhouse gasses, such as CO_2_, CH_4_ and N_2_O; globally elevated temperatures; and changed precipitation distributions that lead to frequent and intense extreme weather events; these conditions causes deficits in soil water availability, reducing plant yields and degrading natural ecosystems (Florides and Paul 2009; Cheng *et al.* 2010). According to the United Nations Intergovernmental Panel on Climate Change, the mean global temperature has risen by ~0.76 °C since 1850 and may further rise by 1.88–4.08 °C by the end of the century without policies to reduce greenhouse gas emission (IPCC 2007).

Temperature changes can affect carbon pools and fluxes within these ecosystems and therefore considerably affect the global carbon cycle. A warmer climate can differentially affect gross ecosystem production and ecosystem respiration. Disproportionate increases in carbon uptake can limit the build-up of atmospheric CO_2_, whereas corresponding increases in carbon release will accelerate this process. The warming also causes a substantial global cycle of nitrogen, generally increasing both the availability and mobility of nitrogen over large regions of land (Vitousek *et al.* 1997). Therefore, unprecedented global warming will have profound long-term impacts on terrestrial plants and ecosystems. Responses of terrestrial plants and ecosystems to global warming may feed back to climate change via ecosystem and global carbon cycling (Beier *et al.* 2004).

Grasslands cover ~3.5 billion ha in 2000, and store 20 % of the world’s soil carbon (Ramankutty *et al.* 2008). Therefore, people significantly rely upon grassland for food and forage (FAO 2009). Global climate change significantly affects grass composition, structure and function through changes in water-use efficiency (WUE), temperature and length of the growing season or by altering the disturbance regimes (Roerink *et al.* 2003; Mu *et al.* 2013; Sun *et al.* 2013). Increased temperatures significantly enhanced the net photosynthetic rate, nutrient availability and lengthening of the growing season; these mechanisms may lead to significant shifts in community structure in a warmer world (Llorens *et al.* 2004; Verlinden *et al.* 2014). Global warming seriously affects the circulation of soil nutrients and the relationships among nutrients, including carbon, nitrogen phosphorus (Zhang *et al.* 2013; Gong *et al.* 2015). Therefore, it is essential to know the distinctive distribution of soil C, N, P and their responses to global warming. The results will contribute to the understanding of soil nutrient status and may have potential significant implications for the management of grassland restoration. *Leymus chinensis* is a perennial rhizome grass widely distributed in the Eastern Eurasian Steppe from North Korea westward to Mongolia and Northern China and north-westward to Siberia (Yang and Li 2003). *Leymus chinensis* accounts for more than two-thirds of the total grassland area in the Songnen Plain in China (Li and Zheng 1997). The use of *L. chinensis* to investigate responses to climate change in the Songnen region will help the development of effective climate change management strategies.

Warming could alter the plant phonological periods, plant community structure, soil physical and chemical properties as well as ecosystem carbon exchange. However, the some results were not consistent because of the time of warming, warming facilities and different environment conditions. As one of the major methodology in global change research, ecosystem warming studies can facilitate model projections on potential changes in terrestrial biomes in terms of parameterization and validation. In this study, we simulated elevated temperatures in the Songnen grassland under otherwise natural conditions, investigated changes in the *L. chinensis* physiology responses to warm climate and analysed *L. chinensis* productivity and conservation of natural grassland ecosystems in response to warm climate change. The results will help ecologists assess and predict the effect of global climate change on major grassland species, such as *L. chinensis*, and provide a basis for grassland management.

## Materials and Methods

### Study site and materials

This research was carried out in Chang Ling County, Jilin Province, China (44°30′–44°45′N, 123°31′–123°56′E). This area has a typical mesothermal monsoon climate with dry, windy spring, hot and arid summers and a mean annual rainfall of 400–500 mm predominantly between June and August. The mean annual temperature in the study area was 5.5 °C, and the accumulated temperatures are 2545.6–3374.2 °C. Evaporation in this area greatly exceeds the mean annual rainfall (Shi and Guo 2006). The soil in Songnen grassland is a soda-saline type, the main cation is Na^+^, and the main anion is HCO_3_^−^ (Qu and Guo 2003). The Songnen grassland is dominated by *L. chinensis*, *Chloris virgata*, *Kalimeris integrifolia*, *Carex duriuscula* and *Rhizoma phragmitis*. The average grass layer height is 80 cm and vegetation coverage is nearly 95 % in the experimental site (Zhang *et al.* 2014).

### Experimental design

**Figure 1.**
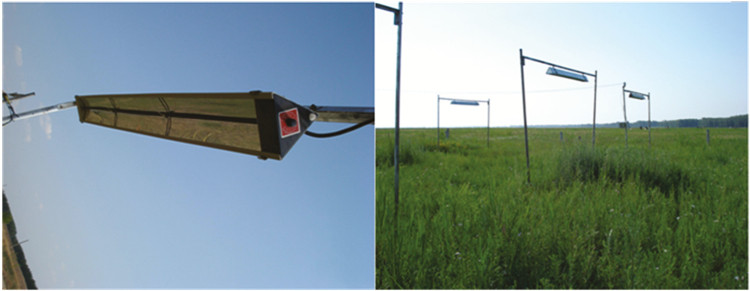
The infrared radiator used to simulate the effects of global warming on the community structures in Songnen grassland in northern China.

The experiment was carried out from April in 2016 to October in 2018 in grassland of the Songnen Plain, and the sites were single dominant *L. chinensis* communities. We set up eight of 3 m × 4 m plots, four warmed plots, and four control plots with 3 m between each plot. The plots shared similar climatic conditions and soil types. The warmed plots were heated continuously with 165 cm × 15 cm MSR-2420 infrared radiators (Kalglo Electronics, Bethlehem, PA, USA) suspended 2.25 m above the ground (Fig. 1) (Wan *et al.* 2002). In experiment plots, the mean month temperature is 24.86 ± 0.36 °C and the month precipitation is 88.1 ± 0.88 mm from June to September in 2018. Each plot represented one replicate in all experiments.

### Measurement of soil properties

Soil temperature and moisture at the depth of 10 cm were measured using an ECH_2_O dielectric aquameter (Em50, USA), which automatically determined the soil temperature and water content in mid-June, mid-July, early August and mid-September. The electrical conductivity (EC) of the soil was measured using a conductivity meter (DDG-2080-S, Anhui, China). The pH was measured using a PSH-3C pH meter (China).

Soil samples were collected in each plot at depths of 0–10, 10–20, 20–30 and 30–40 cm in middle June, July, August and September in 2018. Afterward, the obtained samples were mixed together to obtain a uniform sample and passed through a 0.25-mm soil sieve after natural drying. The soil organic carbon was measured by potassium dichromate heating (Li *et al.* 2014). The content of soil total nitrogen was assayed through Kjeldahl determination (De Borba *et al.* 2014). The content of soil total phosphorus was determined via the sulfuric–perchloric acid method (Bao 2000).

### Measurements of ecosystem gas exchange

Ecosystem gas exchange (CO_2_ and water flux) was measured in a transparent chamber (0.5 m× 0.5 m × 0.9 m) attached to an infrared gas analyser (IRGA; LI-6400, Li-Cor, Lincoln, NE, USA) on one of the two frames in each subplot between 09:00 and 11:00 in the morning. The base of the chamber was sealed while measurements were being carried out, and four small fans ran continuously to mix the air inside the chamber. Nine consecutive readings of CO_2_ and water vapour concentration were recorded on each base at 10-s intervals during a 90-s period. CO_2_ and H_2_O flux rates were determined from the time course of the concentrations and were used to calculate net ecosystem exchange (NEE) and evapotranspiration (ET) rates (Steduto *et al.* 2002). After measurements, the chamber was vented, replaced on the base and covered with an opaque cloth. CO_2_ concentration in the chamber then increased steadily and usually replenished ~30 s after the chamber was covered, at which point the CO_2_ exchange measurements were repeated. Given that the second set of measurements eliminated light (and hence photosynthesis), the values obtained represented ecosystem respiration (ER). Gross ecosystem productivity (GEP) was then calculated as the difference between NEE and ER. Ecosystem WUE was calculated as NEE/ET. Positive NEE values reflect net carbon uptake by the ecosystem, whereas negative values reflect net carbon release.

### Measurement of physiological variables

*Leymus chinensis* was collected carefully from each experimental plot in June, July, August and September in 2018 and then washed with distilled water. The shoots and roots were separated and frozen immediately in liquid nitrogen. The shoots of *L. chinensis* were oven-dried separately at 80 °C and then ground to powder, which was used for physiological index measurements.

Sugar contents were determined by high-performance liquid chromatography (Shimadu, Kyoto, Japan) with Sugar-Pak I columns (Waters Company, Tokyo, Japan). The mobile phase comprised 0.1 mmol L^−1^ EDTA·Na_2_Ca, and the separation was carried out at 70 °C at a flow rate of 0.3 mL min^−1^. The retention times of sucrose, glucose and fructose were 13.545, 16.740 and 20.349 min, respectively. The sucrose, glucose and fructose contents were calculated according to the chromatographic peak area (Rovio *et al.* 2008). The contents of citric acid, malic acid and succinic acid were determined by ion chromatography (DX-300 ion chromatographic system) with ICE-AS6 ion-exclusion column.

The concentrations of ions (Na^+^, K^+^, Ca^2+^ and Mg^2+^) were determined using an atomic absorption spectrophotometer (TAS-990, Purkinje General, Beijing). The concentrations of anions (NO_3_^−^, Cl^−^, SO_4_^2−^ and H_2_PO_4_^−^) were determined using ion-exchange chromatography (DX-300 ion chromatographic system, AS4A-SC ion-exchange column and CD M-II electrical conductivity detector; DIONEX, Sunnyvale, CA, USA) with a mobile phase comprising 1.7 mM Na_2_CO_3_ and 1.8 mM NaHCO_3_ (Yang *et al.* 2007).

### Statistical analysis

Statistical analysis was conducted by repeated measures ANOVA to examine warming effects on soil microclimate, ecosystem C, water fluxes, physiological variables and ions over the growing season in 2018. Between-subject effects were evaluated as warming treatments; within-subject effects were time-of-season. One‐way ANOVA was used to examine the statistical difference in averages for measuring variables among the four months (June, July, August and September). All statistical analyses were conducted with SPSS software (SPSS 19.0 for WINDOWS, Chicago, IL, USA) and standard errors of four replicates.

## Results

### Measurement of soil properties

Trends in the changes in soil properties under the two treatment conditions were similar, but the change under warming condition was greater than that under ambient condition (Table 1). Experimental warming manipulated with infrared radiators significantly elevated soil temperature (Table 1). Across the whole experimental period, under warming mean soil temperature at 10 cm depth was 1.76 °C higher than ambient condition (Table 1). However, warming decreased seasonal mean soil moisture content significantly as compare with ambient condition (Table 1. There was no significant difference in the EC and pH between treatments (Table 1). Compared with ambient condition, warming showed almost no effect on the contents of soil total P, but soil organic C and soil total N content was increased dramatically (Table 1).

**Table 1.** Effects of experimental warming on the growing season mean soil properties of soil temper true (ST), soil moisture (SM), electrical conductivity (EC), pH, soil organic C (SOC), soil total N (STN) and soil total P content (STP). Significant differences between CK and W plots were determined by ANOVA and marked as ‘*’ (*P* < 0.05). CK, ambient plots; W, warming plots.

Plots/parameters	Growing season mean soil properties						
	ST (°C)	SM (v/v %)	EC (dS m^−1^)	pH	SOC (g kg^−1^)	STN (g kg^−1^)	STP (g kg^−1^)
CK	0.94 ± 0.32	22.56 ± 4.77*	1.12 ± 0.44	8.42 ± 1.62	10.78 ± 0.69	0.21 ± 0.02	0.38 ± 0.03
W	1.76 ± 0.58*	20.85 ± 5.13	1.26 ± 0.38	8.34 ± 2.21	12.25 ± 0.72*	0.27 ± 0.02*	0.38 ± 0.03

### The flux of carbon, nitrogen and WUE

**Figure 2.**
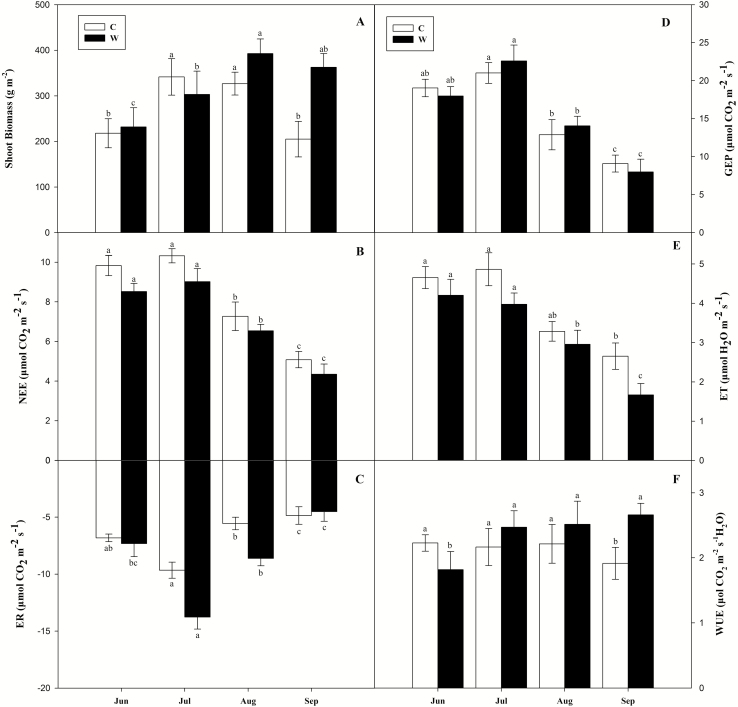
Seasonal dynamics and means (mean ± SE) of (A) shoot biomass, (B) net ecosystem CO_2_ exchange (NEE), (C) ecosystem respiration (ER), (D) gross ecosystem productivity (GEP), (E) ecosystem evapotranspiration (ET) and (F) ecosystem water-use efficiency (WUE) in experiments plots. CK, ambient plots; W, warming plots. One-way ANOVA was used to examine the statistical difference in averages for measuring variables among the four months (June, July, August and September). Different letters in figures indicate significant difference (*P* < 0. 05) in seasonal averages for measuring variables among the four months.

The shoot biomass showed a peak reflecting seasonal dynamics in all plots, but the mean shoot biomass in the warmed plots (322.76 g m^−2^) was higher than that in the ambient plots (272.93 g m^−2^) throughout the growing season (Fig. 2A; Table 2). The season patterns of NEE, ER, GEP and ET were similar and could be expressed a single peak curve. The maximum values appeared in July (Fig. 2B–E). In the control plots, the seasonal mean NEE (8.13 µmol m^−2^ s^−1^) and ET (3.86 µmol m^−2^ s^−1^) values were 14.35 and 17.10 % higher than those in the warmed plots, whereas ER (−6.72 µmol m^−2^ s^−1^) was 27.38 % lower (Fig. 2; Table 2). Gross ecosystem productivity was not significantly different between the two experiment plots (Fig. 2; Table 2). The temporal dynamics of WUE was not obvious, and the low value appeared in September and June in ambient and warmed plots, respectively (Fig. 2F). The seasonal mean WUE (2.06 µmol m^−2^ s^−1^) in the control plots was 32.04 % lower than that in the warmed plots (2.72 µmol m^−2^ s^−1^; Table 2).

**Table 2.** Effect of warming on the seasonal averages of shoot biomass (SB), net ecosystem CO_2_ exchange (NEE), ecosystem respiration (ER), gross ecosystem productivity (GEP), ecosystem evapotranspiration (ET) and ecosystem water-use efficiency (WUE) values. Significant differences between CK and W plots were determined by ANOVA and marked as ‘*’ (*P* < 0.05). CK, ambient plots; W, warming plots.

Plots/parameters	Growing seasonal means of biomass and ecosystem gas exchange parameters					
	SB (g m^−2^)	NEE (µmol m^−2^ s^−1^)	ER (µmol m^−2^ s^−1^)	GEP (µmol m^−2^ s^−1^)	ET (µmol m^−2^s^−1^)	WUE (µmol m^−2^ s^−1^)
CK	272.93 ± 34.00	8.13 ± 0.55*	−6.72 ± −0.59	15.50 ± 1.40	3.86 ± 0.03*	2.06 ± 0.25
W	322.76 ± 41.25*	7.11 ± 0.48	−8.56 ± −0.93*	15.65 ± 1.57	3.20 ± 0.40	2.72 ± 0.34*

### Measurement of sugar and organic acid contents

**Figure 3.**
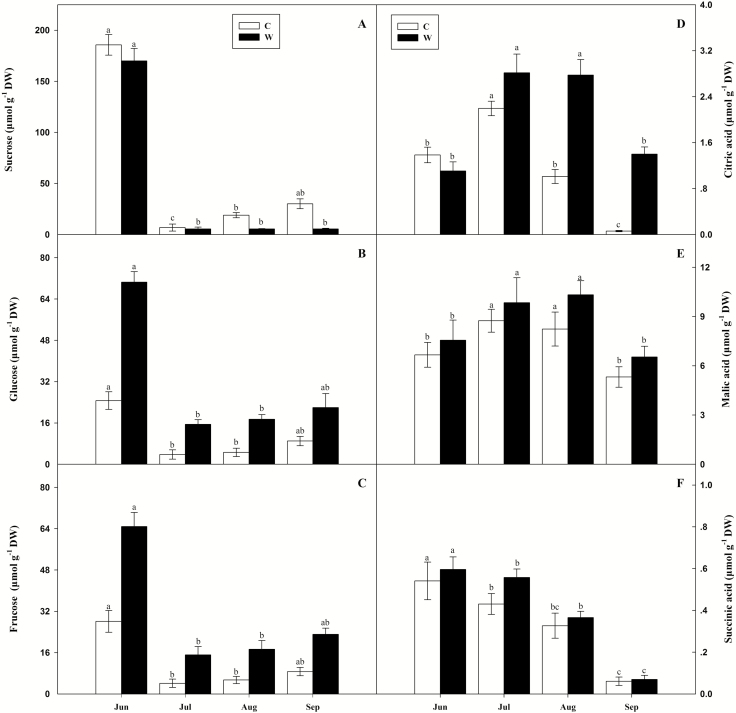
Seasonal dynamics and means (mean ± SE) of sucrose (A), glucose (B), fructose (C), citric acid (D), malic acid (E) and succinic acid (F) in shoots in experiment plots. CK, ambient plots; W, warming plots. One‐way ANOVA was used to examine the statistical difference in averages for measuring variables among the four months (June, July, August and September). Different letters in figures indicate significant difference (*P* < 0. 05) in seasonal averages for measuring variables among the four months.

We have identified sucrose as the major sugar in *L. chinensis* shoots (Table 3). Compared with ambient plots, the warmed plots showed significantly decreased sucrose contents but dramatically increased glucose and fructose contents (Table 3). In seasonal dynamics, the change trends of sucrose, glucose and fructose were similar, and their maximum value appeared in June and then decreased (Fig. 3A–C). Citric and malic acid contents increased under warming condition, but elevated temperature appeared to not affect succinic acid contents of shoot (Table 3). Citric and malic acid contents increased in July and then decreased during the rest of the season, whereas the contents of succinic acid fell over the same period (Fig. 3D–F).

**Table 3.** Effect of warming on the seasonal averages of sucrose, glucose, fructose, citric acid, malic acid and succinic acid. Significant differences between CK and W plots are determined by ANOVA and marked as ‘*’ (*P* < 0.05). CK, ambient plots; W, warming plots.

Plots/parameters	Sugars (µmol g^−1^ DW)			Organic acids (µmol g^−1^ DW)		
	Sucrose	Glucose	Fructose	Citric acid	Malic acid	Succinic acid
CK	241.60 ± 20.93*	42.05 ± 8.60	46.30 ± 8.82	4.65 ± 0.39	28.96 ± 3.11	1.36 ± 0.22
W	186.39 ± 15.07	125.43 ± 13.03*	120.35 ± 14.42*	8.10 ± 0.88*	34.27 ± 4.26*	1.59 ± 0.15

### Measurement of cation and anion concentrations

In response to warming, the percentages of K^+^, Ca^2+^ and Mg^2+^ in the shoots increased, whereas Na^+^ content decreased significantly. However, warming had no significant effect on the levels of the anions Cu^2+^, Fe^2+^, Zn^2+^ and Mn^2+^ and the cations Cl^−^, NO_3_^−^, H_2_PO_4_^−^ and SO_4_^2−^ (Table 4).

**Table 4.** Percentage of the contribution of various free ions to total charge on the seasonal averages of *L. chinensis* shoots tissue in ambient and warmed plots. Significant differences between CK and W plots were determined by ANOVA and marked as ‘*’ (*P* < 0.05). CK, ambient plots; W, warming plots.

Plots/parameters	Anion (%)								Cation (%)			
	K^+^	Na^+^	Ca^2+^	Mg^2+^	Cu^2+^	Fe^2+^	Zn^2+^	Mn^2+^	Cl^−^	NO_3_^−^	H_2_PO_4_^−^	SO_4_^2−^
CK	4.87	20.56*	7.64	7.57	0.87	0.77	0.06	0.06	40.57	0.83	9.59	6.62
W	6.32*	15.41	8.98*	8.83*	0.96	0.91	0.08	0.06	41.23	0.88	9.55	6.79

## Discussion

### Soil organic carbon, N and P in response to manipulations of ambient and warm conditions

The unprecedented global warming will have profound long-term impacts on terrestrial plants and ecosystems (Trumbore 1997). Responses of terrestrial plants and ecosystems to global warming may feed back to climate change via ecosystem and global carbon cycling. Soil organic carbon is the active part in soil and is a crucial component of the earth’s carbon; its storage and fluxes play a major role in the global budget of carbon (Mohan *et al.* 1999). Our results showed that the maximum value of soil organic C appeared in July, and the mean value in growing seasonal under warming treatment was higher by 12.02 % than in ambient condition (Table 1). Combined shoot biomass results in Table 2, implying elevated temperature could stimulate plant activities, meanwhile, soil organic C will increase for more carbon input from plant litter (Zhou *et al.* 2007). Our results also showed that compared with ambient condition, warming condition increased the content of soil total N, mainly because warming is associated with vigorous seasonal vegetation growth that increases the shoot biomass and the absorption of the nutrients is enhanced. Therefore, large accumulations of available N in soil after mineralization when warming enhanced the rate of microbial activity; a similar result was also reported by Piatek and Allen (1999) and Knoepp and Swank (2002). In the current study, we found that during the growing season, mean soil total P was not affected by warming treatment, though shoot biomass increased need more available P. This phenomenon was probably due to the increase in soil microbial activity that are related to decomposed the litter. A large amount of P is returned to the soil, and the amount of total P is not much changed (Chen *et al.* 2003).

### Effects of elevated temperature on carbon and water fluxes

Warming is an important indicator of global climate change and strongly influences the physiological and ecological characteristics of plants (Billings *et al.* 1982; Zhou *et al.* 2007). Our results have shown that elevated temperature increased biomass accumulation in *L. chinensis*, resulting in increased length and thickness of stems; this condition may also induce the phonological process, stimulating root and microbial respiration (Billings *et al.* 1982; Knoepp and Swank 2002; Zhou *et al.* 2007). Therefore, warming induced a significant increase in species richness in the studied meadow steppe community, which is in accordance with the results observed in temperate grasslands and annual grassland (Zavaleta *et al.* 2010; Yang *et al.* 2011). In the midsummer months, when the rate of vegetation growth was greatest, the NEE, GEP and ER rates reached their maximum values. Later in the growing season, GEP and ER tended to decrease as senescence occurred and air temperature declined (Fig. 2). Transpiration and soil evaporation also increased with air and soil temperature in spring and early summer, leading to an increase in ET (Fig. 2E). The senescence of plant tissue and falling soil temperature in late autumn generally caused ET decline. Our data concerning seasonal carbon and water flux in the Songnen grassland in Northeastern China were comparable with those reported in other temperate grasslands (Niu *et al.* 2008). Moreover, our NEE values were similar to those reported for other grassland ecosystems (Flanagan *et al.* 2002). In this experimental, warming increased ER more than GEP, leading to a decrease in NEE. It implied that warming effect on NEE could be largely attributable to ER, and consequently lower soil moisture. In the other way, warming increased WUE could stimulate root and microbial activities and respiration and the direct positive temperature effect, leading to insignificant changes in GEP. The results also suggested that plant transpiration accounted for the majority of ecosystem ET in the Songnen grassland. A similar result was reported for semiarid grassland in North America and temperate steppes in China (Niu *et al.* 2008).

### Effects of elevated temperature on sugars and organic acids

The *L. chinensis* stems were long and thick in warming plots, and their formation depends on the accumulative properties of cellulose during fibre thickening development. The accumulation of cellulose is a process related to the decomposition and recycling of sucrose, glucose and fructose sugars; the process is affected by related substances and enzymes (Winter and Huber 2000; Babb and Haigler 2001). Figure 3 shows that sugar contents in *L. chinensis* declined throughout the growing season, particularly in July. Sucrose is the main storage sugar in *L. chinensis*. Sucrose contents were lower in plants subjected to warming than that in ambient plots. However, glucose and fructose contents were markedly higher in warming than ambient conditions. Our results indicated that warming might have prompted the sustained increased activities of sucrose synthase and sucrose phosphate synthase, whereas the activity of acid invertase and alkaline invertase decreased, and the sucrose transformed thoroughly (Candace *et al.* 2001; Saxena and Brown 2000). Thus, glucose and fructose content was increased, and cellulose accumulated sustainably and steadily, resulting in long and thick stems. The results showed that the elevated temperature induced increases of tricarboxylic acid cycle (*TCA* cycle) intermediates citric acid, malic acid and succinic acid, supporting the notion that *TCA* cycle processes promotion, probably through warming effects. Organic acids are known to play important roles in osmotic adjustment. Organic acids accumulated in the cytoplasm of *L. chinensis* cells in warm conditions, helping to balance the osmotic pressure in the vacuoles. This feature may represent a key adaptive mechanism by which *L. chinensis* maintains homeostasis at elevated temperatures. The results suggest that elevated temperatures caused mass dissipation of energy and enhanced sugar synthesis.

### Effects of elevated temperature on ionic balance

The cytoplasm of plant cells usually has low Na^+^ content and high K^+^ level to maintain essential enzymatic processes (Munns and Tester 2008). Our results confirmed that competitive relationships exist between K^+^ and Na^+^ during their uptake under the conditions of warming. The percentage of Na^+^ increased, whereas the K^+^ percentage decreased in shoots of *L. chinensis*. Ca^2+^ and Mg^2+^ are necessary to maintain membrane stability and are important components of cell walls and chlorophyll (Philip and Martin 2003; Parida and Das 2005). The responses of Ca^2+^ and Mg^2+^ to elevated temperatures were similar, and their percentages were enhanced in shoots, implying that warming enhanced chlorophyll synthesis and photosynthesis. Thus, warming has a certain adaptation and protection mechanism. The percentages of Cu^2+^, Fe^2+^, Zn^2+^ and Mn^2+^ to the total ions present were very low. Cl^−^, NO_3_^−^, H_2_PO_4_^−^ and SO_4_^2−^ contents increased in *L. chinensis* plants subjected to warming to balance Na^+^ influx. This finding suggests that warming may enhance the uptake of anions from the environment.

## Conclusion

Simulated global warming increased the content of soil organic C and soil total N but did not influence soil total P in the growing season. We found that the effect of the warming treatment significantly increased the levels of biomass stem fresh weight and stem quality of *L. chinensis*, whereas experimental warming increased ER, leading to a decrease in NEE and ET. We supposed that the measured C fluxes reduced might be attributed to offsetting of the direct and positive effects of elevated temperature by the indirect and negative effects via exacerbating osmotic stress. On the other hand, warming induced metabolic changes in sugar synthesis. The enhanced formation of sugars was established probably as a reaction to attenuate the osmotic stress caused by warming. The active synthesis of organic acids in leaves was essential to adaption of high temperature. To adapt to elevated temperature, *L. chinensis* accumulated K^+^ and reduced Na^+^ to maintain the intracellular ion balance and a number of enzymatic processes.

## Sources of Funding

This work was supported by the Project of the National Natural Science Foundation of China (grant no. 31570328).

## Contributions by the Authors

R.G. designed the research. J.Z. and R.G. performed the research. X.L.Z., F.X.G. and R.G. analysed the data, and R.G., J.Z., X.L.Z., F.X.G., Q.L. and H.R.L. wrote the paper. All authors reviewed the manuscript.

## Conflict of interest

None declared.
